# Opposite Roles of Tumor Cell Proliferation and Immune Cell Infiltration in Postoperative Liver Metastasis of PDAC

**DOI:** 10.3389/fcell.2021.714718

**Published:** 2021-08-16

**Authors:** Guangfu Wang, Shangnan Dai, Hao Gao, Yong Gao, Lingdi Yin, Kai Zhang, Xumin Huang, Zipeng Lu, Yi Miao

**Affiliations:** ^1^Pancreas Center, First Affiliated Hospital of Nanjing Medical University, Nanjing, China; ^2^Pancreas Institute, Nanjing Medical University, Nanjing, China; ^3^Pancreas Center, The Affiliated BenQ Hospital of Nanjing Medical University, Nanjing, China

**Keywords:** pancreatic cancer, liver metastasis, weighted gene co-expression network analyses, immune cell infiltration, cell proliferation

## Abstract

**Background:**

Recurrence of liver metastasis after pancreatectomy is often a predictor of poor prognosis. Comprehensive genomic analysis may contribute to a better understanding of the molecular mechanisms of postoperative liver metastasis and provide new therapeutic targets.

**Methods:**

A total of 67 patients from The Cancer Genome Atlas (TCGA) were included in this study. We analyzed differentially expressed genes (DEGs) by R package “DESeq2.” Weighted gene co-expression network analysis (WGCNA) was applied to investigate the key modules and hub genes. Immunohistochemistry was used to analyze tumor cell proliferation index and CD4^+^ T cells infiltration.

**Results:**

Functional analysis of DEGs between the liver metastatic and recurrence-free groups was mainly concentrated in the immune response. The liver metastasis group had lower immune and stroma scores and a higher TP53 mutation rate. WGCNA showed that the genes in key modules related to disease-free survival (DFS) and overall survival (OS) were mainly enriched in the cell proliferation process and tumor immune response. Immunohistochemical analysis showed that the pancreatic cancer cells of patients with early postoperative liver metastasis had higher proliferative activity, while the infiltration of CD4^+^ T cells in tumor specimens was less.

**Conclusion:**

Our study suggested that increased immune cell infiltration (especially CD4^+^ T cells) and tumor cell proliferation may play an opposite role in liver metastasis recurrence after pancreatic cancer.

## Introduction

Pancreatic ductal adenocarcinoma (PDAC) is the fourth leading cause of cancer-related death in the United States (Siegel et al., 2021). Surgical resection is the only curative treatment for PDAC. However, the poor postoperative prognosis is usually common for most patients because of the locoregional recurrence and distant metastasis (Mizrahi et al., 2020). Several studies have investigated recurrence after curative resection of PDAC. And more than half of patients with pancreatic cancer develop hepatic recurrences after surgery, according to the literature (Sperti et al., 1997; Van den Broeck et al., 2009). In another study, which evaluated 692 patients with comprehensive and detailed follow-up data, more than a quarter of patients had only liver metastasis after surgery, with a median duration of 6.9 months. Meanwhile, the author also found that the lung-only recurrence occurred later, with a median time of 18.6 months (Groot et al., 2018b). Similar results were confirmed in another study, suggesting that early liver metastasis after surgery is a risk factor for shorter survival in pancreatic cancer patients (Kolbeinsson et al., 2020). Thus, it indicated that postoperative liver metastasis (especially liver-only recurrence) is one of the reasons for the poor prognosis of pancreatic cancer patients after surgery. Moreover, the molecular phenotype and biological behavior of tumors may vary significantly among different types of metastases.

As a result, accurate prediction of the risk of postoperative liver metastasis and identification of therapeutic targets are of great significance for guiding the postoperative treatment of pancreatic cancer, and gene-level analysis can be the most profound interpretation of the molecular characteristics associated with liver metastasis. Therefore, the purpose of this study was to investigate the possible molecular mechanisms of postoperative liver metastasis of pancreatic cancer to find potential molecular targets.

## Materials and Methods

### Acquisition of Datasets

**FIGURE 1:**
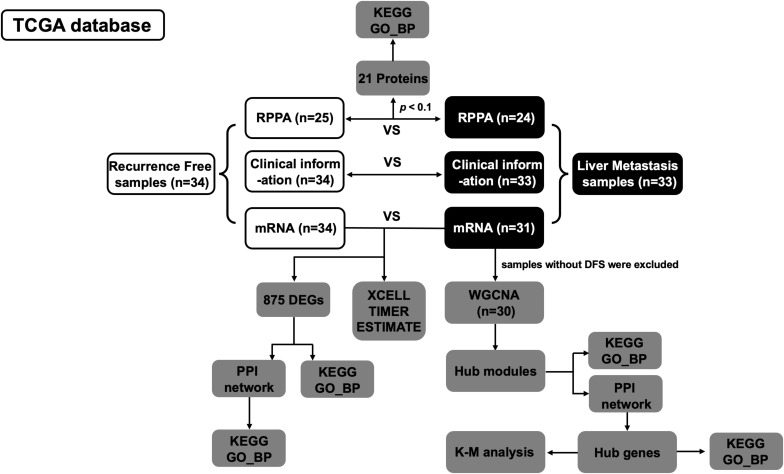
Flowchart of data acquisition, processing, and analysis.

Clinical information (*n* = 67) of PDAC samples and corresponding mRNA sequencing data (*n* = 65) were downloaded from The Cancer Genome Atlas (TCGA).^1^ In addition, normalized reverse phase protein array (RPPA) data were obtained from cBioPortal,^2^ which measured 218 proteins in 49 out of 67 PDAC samples (Figure 1).

### Patients and Samples

Twenty-seven patients who underwent pancreatectomy for PDAC in our center between June 2015 and December 2016 were considered suitable for this study. By the last follow-up, 13 patients had liver metastases recurrence (disease-free survival (DFS) of 10 patients was less than 12 months, and the other 3 patients had a DFS of 13, 15, and 22 months, respectively), while 14 patients had no recurrence (follow-up time of these patients was no less than 36 months). There were no significant differences in age, sex, smoking, alcohol consumption, diabetes mellitus, TNM stage, tumor weight, positive lymph nodes, maximum tumor dimension, pathological stage, and postoperative chemotherapy between the two groups (Table 1). Surgically resected specimens were fixed with 10% formalin and cut into 5 mm thick continuous sections for subsequent studies. This study was approved by the Ethics Committee of the First Affiliated Hospital of Nanjing Medical University.

**TABLE 1:** Patient baseline characteristics.

	**Tumor free (*n* = 14)**	**Liver metastasis (*n* = 13)**	***P* value**
**Age**			
<65	11	9	0.6776
≥65	3	4	
**Sex**			
Male	5	8	0.2568
Female	9	5	
**Alcohol**			
Yes	3	2	0.8096
No	9	8	
NA	2	3	
**Smoking**			
Yes	3	3	0.8132
No	9	7	
NA	2	3	
**DM**			
Yes	2	3	0.6483
No	12	10	
**Residual tumor**			
R0	11	8	0.4197
R1	3	5	
**Grade**			
G1 + G2	7	2	0.1032
G3 + G4	7	11	
**MTD (cm, mean ± SD)**	3.093 ± 1.282 1.203	3.108 ± 1.112	0.9747
**Positive LN (mean ± SD)**	1.214 ± 2.259	1.846 ± 1.864	0.4375
**Stage**			
Stage I + II	13	10	0.3259
Stage III + IV	1	3	
**TMN stage**			
**T stage**			
T1 + T2	13	12	>0.9999
T3 + T4	1	1	
**N stage**			
N0	10	5	0.2268
N1	3	6	
N2	1	2	
**M stage**			
M0	14	13	>0.9999
M1	0	0	
**Post-Chemo**			
Yes	11	8	0.4197
No	3	5	

*DM, diabetes mellitus; MTD, maximum tumor dimension; LN, lymph node; Post-Chemo, postoperative chemotherapy.*

### Immunohistochemical Analysis

Paraffin-embedded tissue from pancreatic cancer patients was processed using standard methodology. Tissue samples were deparaffinized and rehydrated, and antigen retrieval was performed by citrate buffer (pH 6) for 15 min at 95°C. Slides were incubated overnight with primary antibody anti-PCNA (1:200, 60097-1-Ig; ProteinTech, China) and anti-CD4 (1:100, ab133616; Abcam, United Kingdom) at 4°C. The appropriate amount of secondary antibody (1:100; Beyotime, China) was added and incubated at room temperature for 2 h. Diaminobenzidine (DAB) substrate was used for color development. Slides were counterstained with Mayer’s hematoxylin for 1 min.

### Identification of Differentially Expressed Genes

The R package “DESeq2” was used to identify the differentially expressed genes (DEGs). Adjusted *p*-value <0.05 and | Log2fold change| >1 were set as cut-off criteria.

### Construction of Weighted Gene Co-expression Networks

The mRNA sequencing data from 30 postoperative liver metastasis patients were used to construct the weighted gene co-expression network by WGCNA package in R software (version 4.0.2). First, we set the soft threshold as 6 (scale-free *R*^2^ = 0.85) and establish an adjacent matrix according to the connectivity degree so that our genes can satisfy the precondition of scale-free network distribution. Then, the adjacency matrix was transformed into a topological overlap matrix (TOM), which was used to describe the similarity of gene expression, and 1-TOM represented the inter-gene heterogeneity. The TOM is clustered by dissimilarity between genes, and then the tree is cut into different modules by a dynamic tree cutting algorithm. Here, we set the cut height at 0.25 and the minimum module size to 30. Module eigengene (ME) of each module is calculated as the first principal component of a specific module.

### Identification of Clinically Relevant Modules and Key Module Genes

We calculated the correlation between the clinical traits and the MEs through the Pearson correlation coefficient, and *p* < 0.05 was considered to be significant. Key module genes were identified in specific modules under the threshold of module membership | MM| >0.80 and gene significance | GS| > 0.2.

### Functional Enrichment Analysis

We used the Kyoto Encyclopedia of Genes and Genomes (KEGG) pathway analysis to reveal the key pathways associated with these DEGs. In addition, Gene Ontology (GO) terms enrichment analysis was used to identify the functions of these DEGs in terms of biological process (BP). The clusterProfiler package in R software conducted the analysis, and *p* < 0.05 was considered to have statistical significance. ggplot2 packages were utilized for the visualization of the identified DEGs.

### Construction of Protein–Protein Interaction Networks

In this study, the STRING database^3^ was applied to construct the PPI network of DEGs and hub genes identified in specific modules. Confidence score >0.9 was set as the minimum required interaction score. Using the cytoHubba plugin based on Cytoscape (version 3.8.0), the top 10 ranked genes were considered as hub genes in the PPI network.

### Identification of Hub Genes and Construction of a Prognostic Signature

We used DFS to represent the time of postoperative recurrence of liver metastasis. A univariate Cox proportional hazards regression analysis was performed to investigate the association between the top 10 ranked genes and DFS and overall survival (OS). *p* < 0.05 was considered as prognostic genes. The prognostic genes were further applied to stepwise multivariate Cox proportional hazards regression analysis to predict the risk value of each patient. The patients were divided into high- and low-risk groups, and the predictive value of these genes was estimated by mapping the receiver operating characteristic (ROC) curve. The sensitivity and specificity of the DFS risk model were evaluated by calculating the area under the ROC curve using the “timeROC” R package.

### Statistical Analysis

The Student’s *t*-test was applied to compare the diverse expressions of PCNA and numbers of CD4^+^ T cells in the recurrence-free and liver metastases recurrence groups in our specimens. Correlation between the immune infiltration level and the DFS and OS was analyzed by using a Pearson correlation test. In addition, Kaplan–Meier curves were conducted to compare the DFS and OS between the hub genes based on the different gene expressions. The statistical analysis was performed using R software (version 4.0.2) and GraphPad Prism (version 8.4.0).

## Results

### Comprehensive Differences Between the Recurrence-Free and Liver Metastasis Groups

#### Patient Baseline Characteristics

A total of 67 patients were enrolled in this study, including 34 recurrence-free patients and 33 liver metastasis patients. There were no significant differences in age, sex, smoking, alcohol consumption, diabetes mellitus, TNM stage, tumor stage, and postoperative chemotherapy between the two groups. However, patients with postoperative liver metastasis had larger tumor maximum dimension trend (3.945 ± 1.012 vs. 3.448 ± 1.065; *p* = 0.0689), higher histopathological grade (*p* = 0.0047), and more positive lymph node number (4.061 ± 3.905 vs. 1.794 ± 2.042; *p* = 0.0039) (Table 2). We also found that patients with postoperative liver metastasis had a higher TP53 mutation rate (*p* = 0.0125) (Table 3).

**TABLE 2:** Patient baseline characteristics from TCGA.

	**Tumor free (*n* = 34)**	**Liver metastasis (*n* = 33)**	***P* value**
**Age**			
<65	15	19	0.3319
≥65	19	14	
**Sex**			
Male	19	16	0.6278
Female	15	17	
**Alcohol**			
Yes	19	19	0.9858
No	12	11	
NA	3	3	
**Smoking**			
Yes	26	28	0.5378
NA	8	5	
**DM**			
Yes	11	6	0.1711
No	16	23	
NA	7	4	
**Residual tumor**			
R0	23	20	0.7402
R1	8	9	
R2	0	1	
NA	3	3	
**Grade**			
G1 + G2	28	16	0.0047*
G3 + G4	6	17	
**MTD (cm, mean ± SD)**	3.448 ± 1.065	3.945 ± 1.012	**0.0689**
**Positive LN (mean ± SD)**	1.794 ± 2.042	4.061 ± 3.905	0.0039*
**Stage**			
Stage I + II	31	33	0.2178
Stage III + IV	2	0	
NA	1	0	
**TMN stage**			
**T stage**			
T1 + T2	3	3	0.6110
T3 + T4	30	30	
NA	1	0	
**N stage**			
N0	11	7	0.4101
N1	23	26	
**M stage**			
M0	33	22	0.2
M1	1	0	
MX	17	11	
**Post-Chemo**			
Yes	27	27	>0.9999
NA	7	6	

DM, diabetes mellitus; MTD, maximum tumor dimension; LN, lymph node; Post-Chemo, postoperative chemotherapy. Bolded values: statistically significant or trend. *P < 0.05.

**TABLE 3:** Patient mutation characteristics from TCGA.

	**Tumor free (*n* = 34)**	**Liver metastasis (*n* = 33)**	***P* value**
**KRAS mutation**
Yes	28	28	0.9999
No	6	5	
**TP53 mutation**
Yes	15	25	0.0125*
No	19	8	
**SMAD4 mutation**
Yes	6	2	0.2585
No	28	31	
**CDKN2A mutation**
Yes	4	5	0.7337
No	30	28	

Bolded values: statistically significant or trend. *P < 0.05.

#### Identification of DEGs and Functional Analysis

**FIGURE 2:**
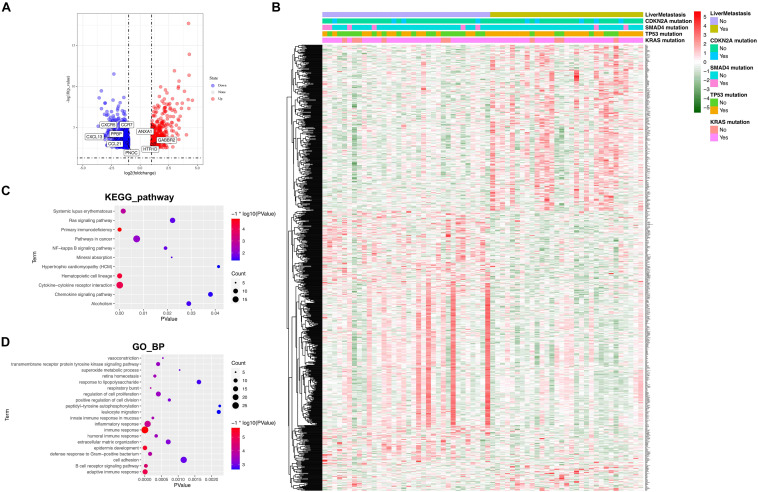
Identification of DGEs and functional analysis. **(A)** The volcano plot for DGEs comparing recurrence free patients with liver metastasis patients. Eight hundred fifty-seven DGEs, including 493 upregulated genes (red dots) and 364 downregulated genes (blue dots), were identified. **(B)** Heatmap of DGEs. Mutation status of KRAS, TP53, SMAD4, and CDKN2A was also attached. **(C)** KEGG pathways analysis of the DGEs. **(D)** GO analysis for biological processes of the DGEs.

Sixty-five patients, including 34 recurrence-free patients and 31 liver metastasis patients with mRNA sequencing data, were subsequently examined, and a total of 857 DGEs were identified (364 downregulated and 493 upregulated). All DGEs were illustrated by volcano plots and heatmap (Figures 2A,B). Then, we annotated functions of DEGs by KEGG pathway and GO analysis for biological processes (GO_BP). KEGG pathway analysis suggested that the DEGs were significantly enriched in “cytokine–cytokine receptor interaction, primary immunodeficiency, hematopoietic cell lineage, systemic lupus erythematosus, and pathway in cancer.” The results of GO_BP analysis showed that DEGs were significantly enriched in “immune response, adaptive immune response, epidermis development, B cell receptor signaling pathway, and inflammatory response” (Figures 2C,D). This enriched KEGG pathway and GO_BP terms could further help us understand functions of DEGs in the development of postoperative liver metastasis.

#### Construction of PPI Network

**FIGURE 3:**
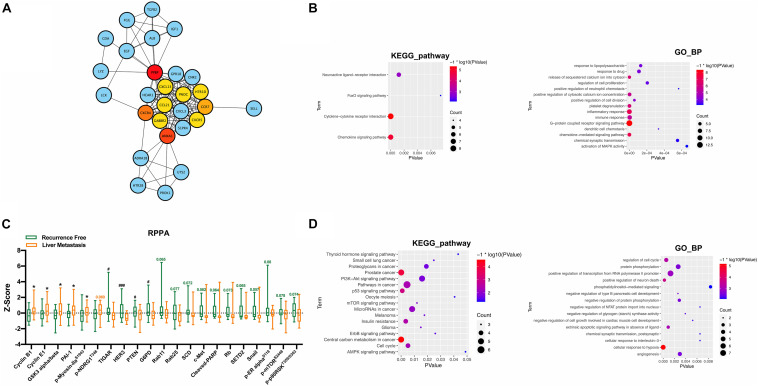
PPI network and RPPA analysis along with functional analysis. **(A)** PPI network of DEGs including hub genes and subnetwork related to hub genes. **(B)** KEGG pathway and GO_BP analysis of recognized 28 genes. **(C)** RPPA analysis between the recurrence free group and the liver metastasis group. **(D)** KEGG pathway and GO_BP analysis of proteins with *p*-value less than 0.1 recognized in the RPPA analysis. ^*,#^*P* < 0.05, ^###^*P* < 0.001.

The PPI network of DEGs was constructed for further analysis, including 460 nodes, 329 edges, and PPI enrichment *p*-value <1.0e^–16^. Using the cytoHubba plugin based on Cytoscape to check the first stage nodes, the top 10 ranked proteins were identified as hub genes, which may play a significant role in the development of postoperative liver metastasis. Moreover, the shortest path and the expanded subnetwork associated with the 10 hub genes were identified (Figure 3A). KEGG pathway and GO_BP analysis were carried out to annotate the 28 functions of genes. KEGG pathway analysis suggested that the function was significantly enriched in the “chemokine signaling pathway, cytokine–cytokine receptor interaction, and neuroactive ligand–receptor interaction.” GO_BP analysis showed significant enrichment in “G-protein coupled receptor signaling pathway, inflammatory response, immune response, and chemokine-mediated signaling pathway” (Figure 3B).

#### RPPA Analysis

The differences in expression levels of specific proteins between the two groups were further analyzed in 49 specimens: 25 in the recurrence-free group and 24 in the liver metastasis group. The results showed that CCNB1, CCNE1, GSK3, PAI-1, and p-Myosin IIa^*S*1943^ were significantly increased in the liver metastasis group, and TIGAR, HER3, PTEN, and G6PD were increased considerably in the recurrence-free group (Figure 3C). Proteins with a *p*-value less than 0.1 were further included in the KEGG pathway and GO_BP analysis. KEGG pathway analysis suggested that the function was significantly enriched in “central carbon metabolism in cancer, prostate cancer, p53 signaling pathway, and pathways in cancer and insulin resistance.” GO_BP analysis showed significant enrichment in “cellular response in hypoxia, positive regulation of neuron death, and regulation of cell cycle” (Figure 3D).

#### Infiltration Level of Immune Cells

**FIGURE 4:**
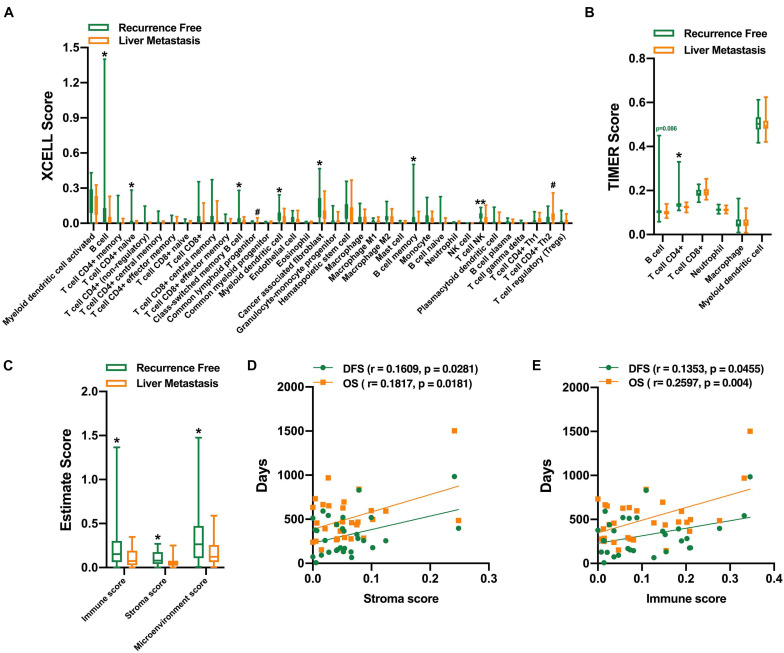
Evaluation of the infiltration level of immune cells. XCELL **(A)**, TIMER **(B)**, and ESTIMATE score of the two groups **(C)**. Positive correlation between stroma score **(D)**, immune score **(E)**, and the DFS and OS in the liver metastasis group. ^*,#^*P* < 0.05, ^**^P < 0.01.

Functional analysis of DEGs suggested that there might be differences in tumor immune microenvironment between the two groups. With the estimation of XCELL, the infiltration levels of B cells, CD4^+^ naive T cells, myeloid dendritic cells, and natural killer T (NKT) cells were higher in the recurrence-free group, and Th2 CD4^+^ T cells, common myeloid progenitor cells, were higher in the liver metastasis group (Figure 4A). With the estimation of TIMER, CD4^+^ T cells were significantly higher in the liver metastasis group. There is no significant difference in the B cells (Figure 4B). We further measured the ESTIMATE score of these two groups, and the results suggested that the recurrence-free group had significantly higher immune and stroma scores (Figure 4C). We did not find a correlation between the ESTIMATE scores with the DFS and OS for 65 patients (Supplementary Figure 1). However, there is a significant correlation between the ESTIMATE scores with the DFS and OS in the liver metastasis group (Figures 4D,E).

### Co-expression Network Modules and Hub Genes Identified by WGCNA

#### Construction of WGCNA and Identification of Key Modules

**FIGURE 5:**
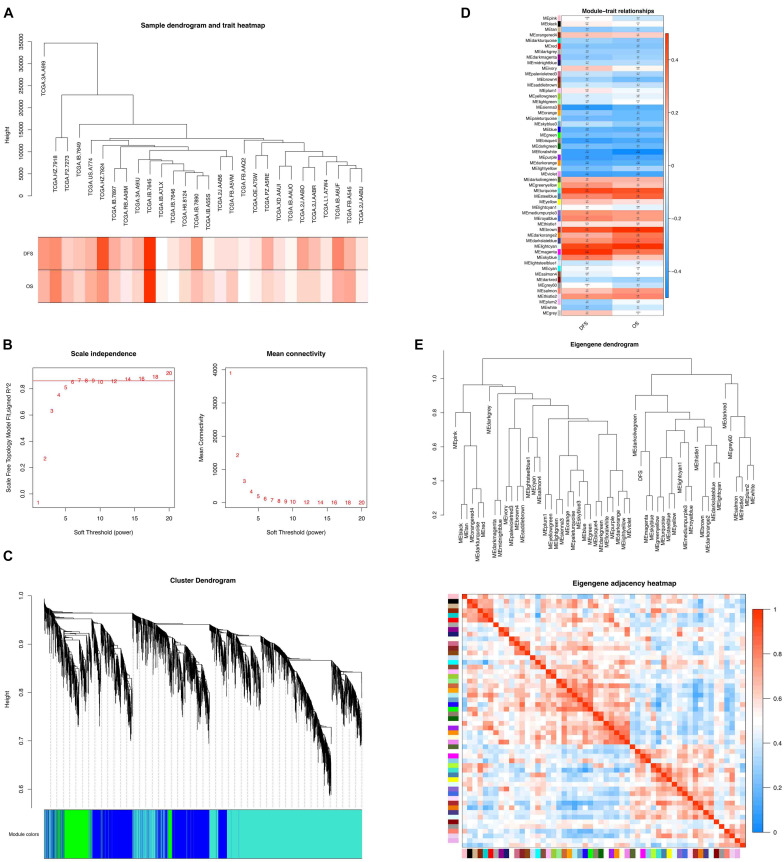
WGCNA network of the liver metastasis group. **(A)** Clustering dendrogram of 28 samples. **(B)** Determination of soft-threshold power in the WGCNA. **(C)** Clustering dendrograms of identified co-expressed genes in modules in the liver metastasis group. Each colored row represents a color-coded module that contains a group of highly connected genes. **(D)** Heatmaps of the correlation between eigengene and DFS and OS of the liver metastasis group. Each row corresponds to an ME, and each cell contains the corresponding correlation (red, positively correlated; blue, negatively correlated) and *p*-value. **(E)** Dendrogram of MEs obtained by WGCNA and heatmap plot of the adjacencies of modules. Red represents high adjacency (positive correlation), and blue represents low adjacency (negative correlation).

Based on the abovementioned studies, we found that the immune response within the tumor and cell cycle may be related to the occurrence of postoperative liver metastasis in pancreatic cancer. To further explore its mechanisms, we constructed the weighted gene co-expression network analysis (WGCNA). WGCNA is a method to find out the significant cluster of genes associated with specific clinical traits. The WGCNA network comprised 30 samples with a specific time for the occurrence of postoperative liver metastasis. Clinical sample information included DFS, time of liver metastasis recurrence, and OS. All samples were clustered, and 28 samples were chosen for further analysis (Figure 5A). The soft threshold was “6” (Figure 5B), and the cluster dendrograms of samples matching the clinical traits were obtained (Figure 5C). Finally, 54 modules were obtained. We found that there were three modules positively correlated with DFS and OS. Also, there were two modules negatively correlated with DFS and OS (Figure 5D). In this study, three modules (turquoise, brown, and purple) were extracted for further analysis because they had the most significant correlation with DFS and OS. Based on the eigengene adjacent heatmap, the three modules showed independent validation to each other (Figure 5E).

#### Functional Analysis of Key Module Genes

**FIGURE 6:**
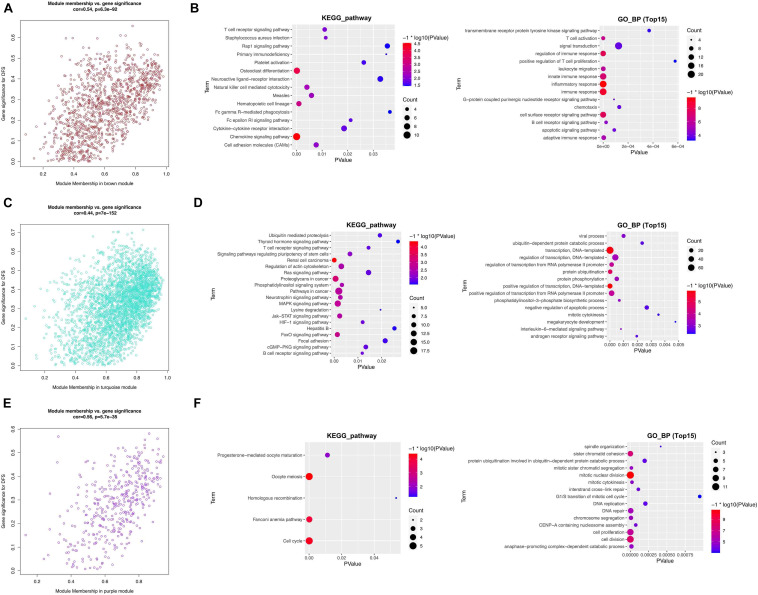
Functional analysis of key module genes. **(A,B)** Brown module and KEGG pathway and GO_BP analysis of key module genes. **(C,D)** Turquoise module and KEGG pathway and GO_BP analysis of key module genes. **(E,F)** Purple module and KEGG pathway and GO_BP analysis of key module genes.

From the module–trait relationship heatmap, the brown module was highly correlated with clinical traits (correlation coefficient = 0.54, *p* = 6.3E^–92^; Figure 6A). The brown module contained 1,202 genes and was correlated with DFS and OS positively. The key module genes were identified in a brown module under the threshold of module membership | MM| >0.85 and gene significance | GS| >0.3. To reveal the functions of these genes, we conduct the KEGG pathway and GO_BP analysis. KEGG pathway analysis suggested that the function was significantly enriched in the “chemokine signaling pathway, osteoclast differentiation, and hematopoietic cell lineage” (Figure 6B). GO_BP analysis exhibited significant enrichment in “inflammatory response, immune response, innate immune response, regulation of immune response, cell surface receptor signaling pathway, and T cell activation” (Figure 6B).

The turquoise module contained 3,205 genes and was correlated with DFS and OS positively (correlation coefficient = 0.44, *p* = 7E^–152^; Figure 6C). The KEGG pathway analysis showed that the function was significantly enriched in “renal cell carcinoma, proteoglycans in cancer, and pathways in cancer” (Figure 6D). GO_BP analysis showed significant enrichment in “transcription, DNA-templated, positive regulation of transcription, DNA-templated, and protein ubiquitination” (Figure 6D).

The purple module contained 407 genes and was correlated with DFS and OS negatively (correlation coefficient = 0.56, *p* = 5.7E^–35^; Figure 6E). The KEGG pathway analysis showed that the function was significantly enriched in the “cell cycle, oocyte meiosis, and Fanconi anemia pathway” (Figure 6F). GO_BP analysis was significantly enriched in “mitotic nuclear division, cell division, cell proliferation, and sister chromatid cohesion” (Figure 6F).

**FIGURE 7:**
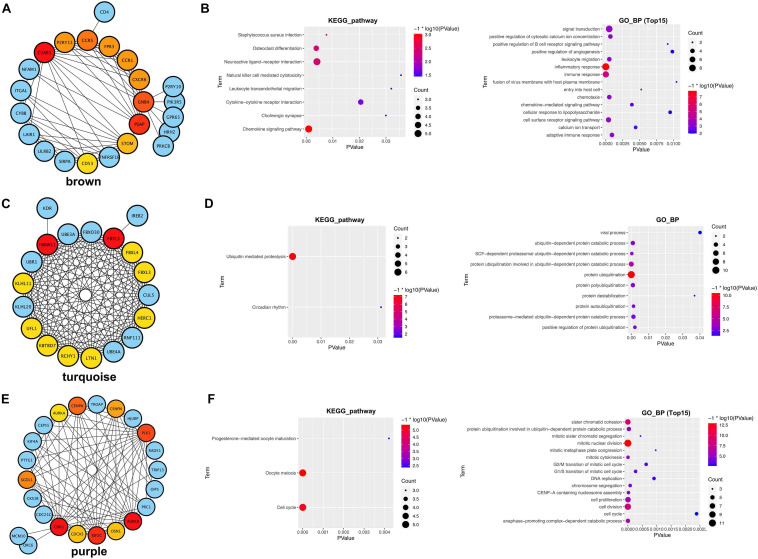
PPI network and functional analysis of three hub modules. **(A,B)** Brown module. **(C,D)** Turquoise module. **(E,F)** Purple module.

We further constructed the PPI network of the three hub modules (Figures 7A,C,E). Moreover, the outcomes of the KEGG pathway and GO_BP analysis of the three module genes are shown in Figures 7B,D,F, respectively.

#### Hub Genes in the Purple Module

**FIGURE 8:**
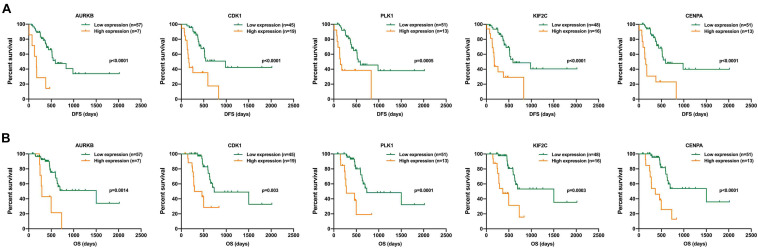
Survival analysis of the top five genes in the purple module. **(A)** OS analysis of the top five genes. **(B)** DFS analysis of the top five genes.

Finally, we found that the purple module was negatively correlated with DFS and OS, and genes CDK1, PLK1, AURKB, CENPN, KIF2C, etc., played the most important role in this module. This suggests that the increased expression of these genes might play an essential role in promoting the occurrence of postoperative liver metastasis of pancreatic cancer. Survival analysis of the top five genes in the purple module showed an inverse relationship between gene expression and prognosis (Figures 8A,B). Meanwhile, we found that the expression of these five genes was elevated in pancreatic cancer (Supplementary Figure 2A). Further analysis in the TCGA database showed that the expression of these five genes was closely related to postoperative recurrence and OS (Supplementary Figures 2B,C).

### Tumor Cell Proliferation, CD4^+^ T Cells Infiltration, and Postoperative Liver Metastasis

**FIGURE 9:**
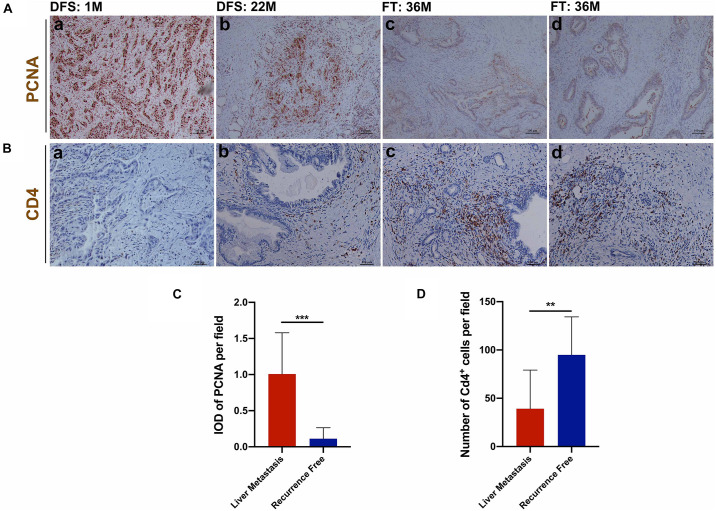
Immunohistochemical analysis of tumor proliferation and CD4^+^ T cells infiltration. **(A)** (a,b) Proliferative activity of tumor cells decreased with the prolongation of liver metastasis; (c,d) Proliferative activity of tumor cells was almost undetectable in recurrence-free patients. **(C)** Patients with early postoperative liver metastasis of pancreatic cancer had higher proliferative activity. **(B,D)** Patients with early postoperative liver metastasis had fewer CD4^+^ T cells infiltration. ^**^P < 0.01, ^***^P < 0.001.

The above analysis indicated that increased cell proliferation could promote the occurrence of postoperative liver metastasis of pancreatic cancer and the increase of CD4^+^ T cells infiltration was more obvious in the group without liver metastasis recurrence. In this study, immunohistochemical analysis showed that the tumor cells of patients with early postoperative liver metastasis of pancreatic cancer had higher proliferative activity (Figure 9C) and less CD4^+^ T cells infiltration (Figure 9D). Moreover, the proliferative activity of tumor cells decreased with the prolongation of liver metastasis (Figure 9A). In addition, we found that most of the tumor cells were quiescent in recurrence-free patients, and that there was a significant infiltration of CD4^+^ T cells around the lesions of the pancreatic duct (follow-up time (FT) >3 years) (Figures 9A,B).

## Discussion

Pancreatic cancer is a malignant tumor of the digestive system. Due to local recurrence and distant metastasis, the postoperative prognosis of most patients is poor. Distinct locations of the first recurrence have unique independent predictive values for OS, which is helpful for stratification of prognosis and treatment decision after diagnosis of recurrence (Groot et al., 2018a). Several studies have reported the recurrence patterns after pancreatectomy for PDAC. Most postoperative patients with pancreatic cancer recurred at isolated distant sites, and liver recurrence tended to occur earlier (Groot et al., 2018b). The patterns of postoperative liver recurrence could be used as a basis for specific surveillance and treatment of patients with pancreatic cancer (Tanaka et al., 2019), which was verified by a meta-analysis including 89 studies with 17,313 patients undergoing PDAC resection. In this study, we presented an integrated view of hepatic recurrence after pancreatectomy at the molecular level.

We examined TCGA gene expression profile to identify potential biomarkers for postoperative liver metastasis of pancreatic cancer. Based on the KEGG analysis of DEGs, we found that DGEs were enriched highly in cytokine–cytokine receptor interaction. GO enrichment analysis showed that DEGs were significantly enriched in immune response and adaptive immune response. Furthermore, the KEGG analysis results revealed that key genes in the PPI network regarding DGEs were mostly enriched in the chemokine signaling pathway. In GO analysis, the enrichment of these genes mainly functioned to G-protein coupled receptor signaling pathway, inflammatory response, and immune response. Muller et al. (2001) validated that chemokines and their receptors play a vital role in determining the direction of tumor cell metastasis. G-protein coupled receptor kinases and receptors are also involved in tumorigenesis and cancer aggressiveness (O’Hayre et al., 2013; Wang et al., 2017; Nogues et al., 2018; Brown and Ganapathy, 2020). The immune response is implicated in tumor progression and metastasis in various ways (Berraondo et al., 2016; O’Sullivan et al., 2019; Riera-Domingo et al., 2020). It can protect the host from virus-induced tumors (Reiche et al., 2004), inhabit the inflammatory environment of tumorigenesis (Ebbin, 1989; Elinav et al., 2013), and identify specific antigens of the tumor to eliminate cancer cells (Medzhitov, 2007; Bommareddy et al., 2018). However, as T cells (Chang et al., 2015), NKT (Keating et al., 2016; Cong et al., 2018), macrophages, and dendritic cells (O’Neill and Pearce, 2016) use glucose to support their effector functions, malignant cells can deprive tumor microenvironment (TME) of glucose, thus blocking effective anticancer immunity (Badur and Metallo, 2018). Pancreatic cancer is characterized by dense fibrotic stroma, which promotes the generation of the immunosuppressive, hypoxic, and nutrient-poor TME (Kamphorst et al., 2015).

Analysis with ESTIMATE score also suggested that the recurrence-free group had significantly higher immune and stroma scores, which showed higher infiltration levels of B cells, CD4^+^ naive T cells, myeloid dendritic cells, and NKT cells. However, Th2 CD4^+^ T cells, common myeloid progenitor cells, were higher in the liver metastasis group. These results imply that the occurrence of liver metastasis after pancreatectomy for PDAC is closely correlated with immune cell infiltration. Solid tumors, namely, breast cancer, renal cell carcinoma, melanoma, ovarian cancer, and gastrointestinal stromal tumors, have been shown to have spontaneous T cell infiltration (Zhang et al., 2003; Kreike et al., 2007; Mahmoud et al., 2011; Azimi et al., 2012; Rusakiewicz et al., 2013). In the TME of ovarian cancer, the ratio of CD8^+^ T cells to Foxp3^+^ regulatory T cells was significantly high, which was associated with a particularly favorable prognosis (Curiel et al., 2004).

Our further WGCNA showed that the brown module was correlated with DFS and OS positively. In addition, we found that the function of key module genes was highly enriched in the chemokine signaling pathway by KEGG pathway analysis and in the inflammatory response and immune response by GO analysis. However, the turquoise module and the purple module were correlated with DFS and OS negatively. The function analysis in the purple module possessed cell cycle in KEGG pathway analysis and mitotic nuclear division, cell division, and cell proliferation in GO analysis. Furthermore, in the purple module, the top five genes (CDK1, PLK1, AURKB, CENPN, and KIF2C) showed an inverse relationship between gene expression and prognosis, and their expression was closely related to postoperative recurrence and OS.

Among the top five genes, PLK1 was reported to be closely correlated with pancreatic cancer. Polo-like kinase 1 (Plk1), a serine/threonine kinase of polo-like kinases family, plays an essential role in cell mitosis, spindle assemblies, DNA damage, and so on (Lens et al., 2010; Strebhardt, 2010). Weichert et al. (2005) have reported that PLK1 was found to be overexpressed in pancreatic neoplasia as early as in pancreatic intraepithelial neoplasia III lesions and invasive pancreatic adenocarcinomas were PLK1, strongly positive in 47.7% of cases. Additionally, a study suggested that PLK1 showed >50% downregulation in gemcitabine sensitive cases and no change in the resistant cases (Jimeno et al., 2010). Correspondingly, inhibition of PLK1 also synergized with gemcitabine in gemcitabine-refractory *in vitro* (Jimeno et al., 2010) and *in vivo* models (Li et al., 2016). Moreover, the combination of PI3K/Akt pathway inhibition and PLK1 depletion can enhance chemosensitivity to gemcitabine in pancreatic cancer (Mao et al., 2018). According to the above results, PLK1 was proposed as a promising target for the treatment of gemcitabine-resistant pancreatic cancer. In a phase I trial, rigosertib, a PLK1 inhibitor, was combined with gemcitabine to treat patients with advanced PDAC (Ma et al., 2012). This phase I study determined the recommended phase II dose (RPTD), and under this, RPTD rigosertib is well tolerated with a toxicity profile similar to the gemcitabine alone. After demonstrating the safety of rigosertib, a phase II/III randomized study was then conducted (O’Neil et al., 2015). This phase II/III randomized study was designed to compare the efficacy and safety of rigosertib plus gemcitabine vs. gemcitabine alone in patients with metastatic pancreatic cancer. However, the combination of rigosertib plus gemcitabine failed to improve prognosis or response compared with gemcitabine alone in patients with metastatic pancreatic adenocarcinoma. The failure may be owed to the inherent heterogeneity of pancreatic cancer, and more comprehensive research regarding this topic is urgently needed. Hence, if overexpression of PLK1 for patients with pancreatic cancer after pancreatectomy was detected, non-gemcitabine chemotherapy would be preferred to avoid potential chemotherapeutic resistance. If gemcitabine-based chemotherapy has to be applied, PLK1 inhibitor could be considered to combine with it, and the resulting combination is safe and theoretically helpful.

## Conclusion

Although we found that reduced immune cell infiltration was strongly associated with postoperative liver metastasis of pancreatic cancer, the internal mechanism still needs further investigation. The direct immune killing effect on tumor cells may be the main reason. Furthermore, previous studies have shown that tumor metastasis is closely related to the epithelial mesenchymal transition (EMT) process of tumor cells rather than tumor cell proliferation. However, our study showed that the proliferation of primary tumor cells was significantly increased in patients with postoperative liver metastasis. Therefore, we hypothesized that some patients with pancreatic cancer already had preclinical liver micro-metastases, which could not be detected by current detection methods. Such micro-metastases showed liver metastasis recurrence at different times after surgery due to the different proliferation abilities of tumor cells. According to our clinical tissue samples, patients with early postoperative liver metastasis had significantly more proliferative primary tumors than patients without recurrence.

## Data Availability Statement

The datasets presented in this study can be found in online repositories. The names of the repository/repositories and accession number(s) can be found in the article/Supplementary Material.

## Ethics Statement

The studies involving human participants were reviewed and approved by the Ethics Committee of the First Affiliated Hospital with Nanjing Medical University. The patients/participants provided their written informed consent to participate in this study.

## Author Contributions

GW designed and drafted the manuscript. SD and HG conducted the analyses. YG, LY, KZ, and XH conducted the literature search. ZL and YM designed, led, supervised the study, and critically revised the manuscript. All authors approved the final draft submitted, read and agreed to the published version of the manuscript.

## Conflict of Interest

The authors declare that the research was conducted in the absence of any commercial or financial relationships that could be construed as a potential conflict of interest.

## Publisher’s Note

All claims expressed in this article are solely those of the authors and do not necessarily represent those of their affiliated organizations, or those of the publisher, the editors and the reviewers. Any product that may be evaluated in this article, or claim that may be made by its manufacturer, is not guaranteed or endorsed by the publisher.

## Footnotes

1
http://cancergenome.nih.gov/

2
https://www.cbioportal.org/

3
https://string-db.org

## Supplementary Material

Supplementary Figure 1 No correlation between the ESTIMATE scores with the DFS and OS for 65 patients.

Supplementary Figure 2 **(A)** Top 5 genes in purple module were upregulated in pancreatic cancer. **(B,C)** Top 5 genes in purple module were correlated with DFS and OS of patients with pancreatic cancer.
