# Supplementary figures and images for: Opposite Roles of Tumor Cell Proliferation and Immune Cell Infiltration in Postoperative Liver Metastasis of PDAC
Source: Front Cell Dev Biol. 2021 Aug 16;9:714718. doi: 10.3389/fcell.2021.714718 (PMC8415276; doi:10.3389/fcell.2021.714718)

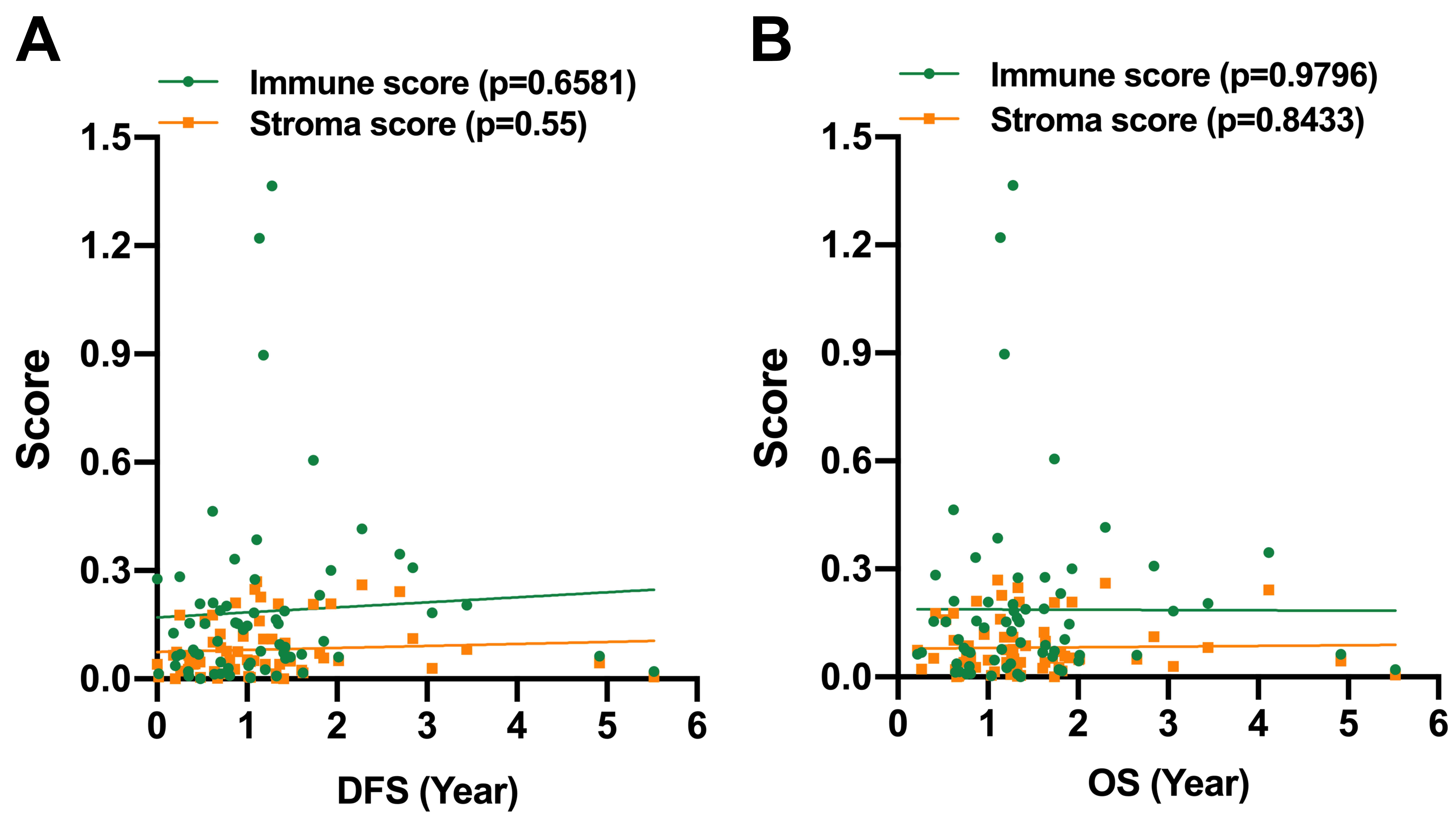

Supplement: Supplementary Figure 1 — No correlation between the ESTIMATE scores with the DFS and OS for 65 patients. [file Image_1.JPEG]
