# Supplementary material for: Opposite Roles of Tumor Cell Proliferation and Immune Cell Infiltration in Postoperative Liver Metastasis of PDAC
Source: Front Cell Dev Biol. 2021 Aug 16;9:714718. doi: 10.3389/fcell.2021.714718 (PMC8415276; doi:10.3389/fcell.2021.714718)
Supplement: Supplementary file 3 [file Data_Sheet_1.docx]

**PCNA:**

**Postoperative Liver Metastasis (n=13)**


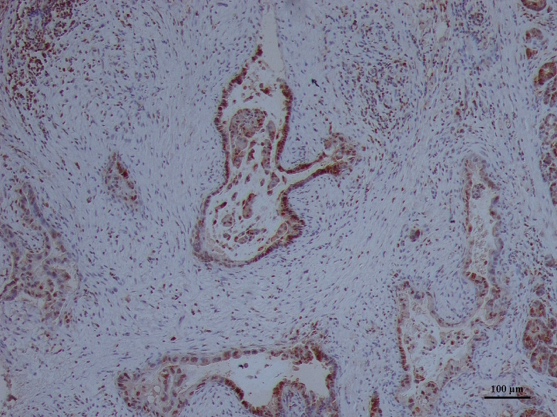

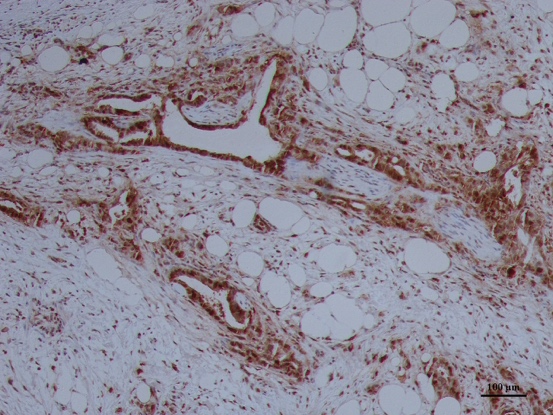


Patient 1 Patient 2


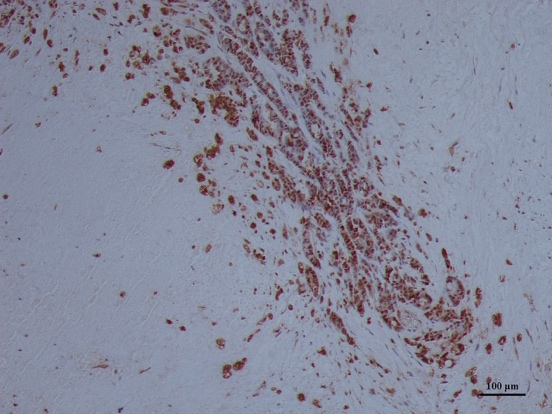

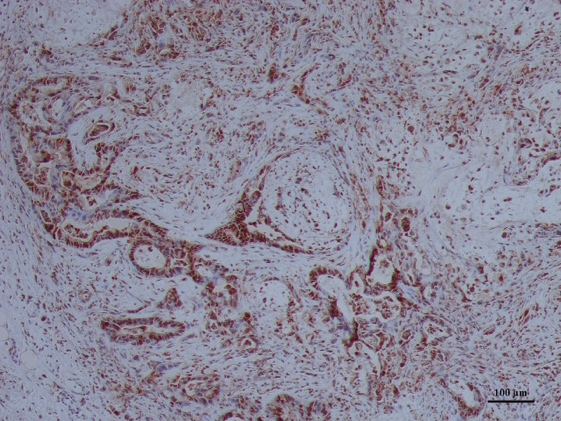


Patient 3 Patient 4


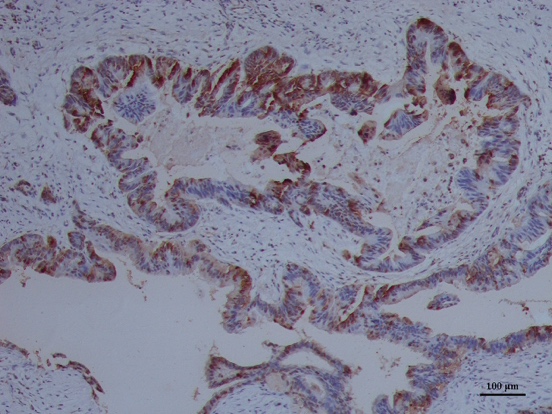

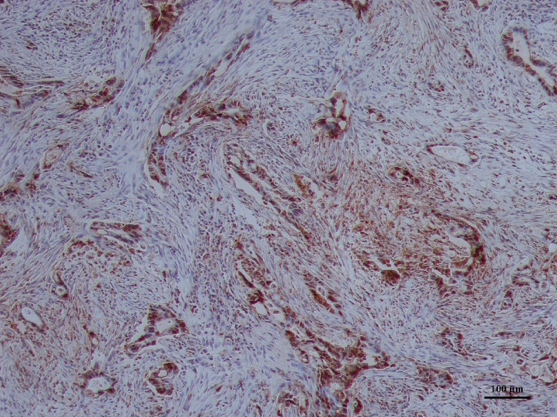


Patient 5 Patient 6


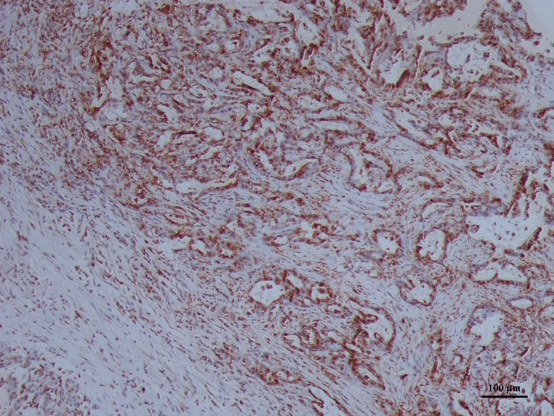

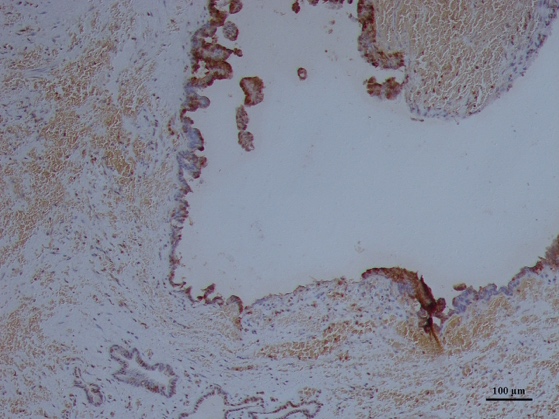


Patient 7 Patient 8


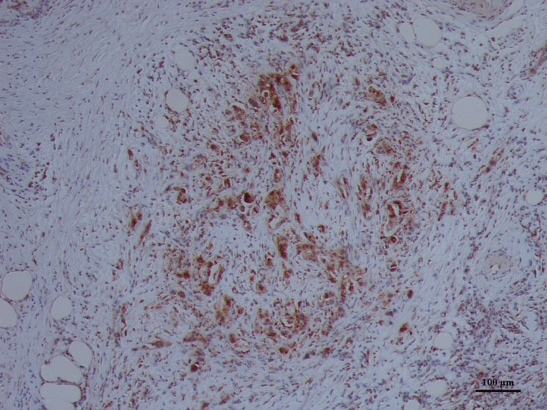

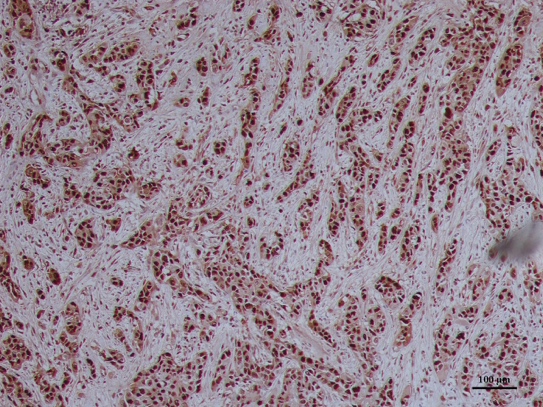


Patient 9 Patient 10


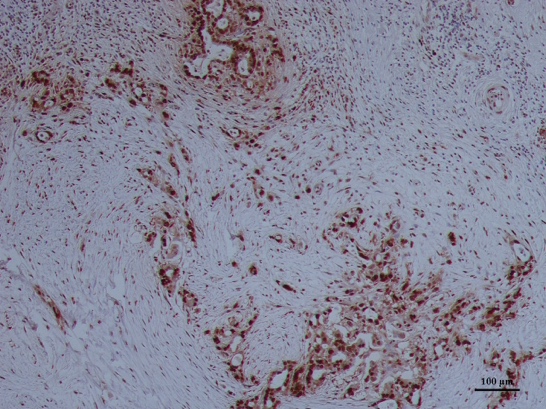

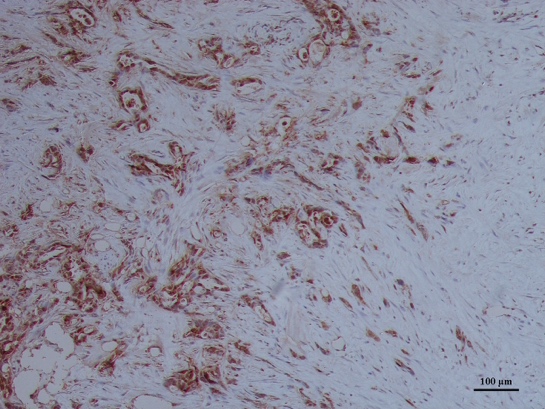


Patient 11 Patient 12


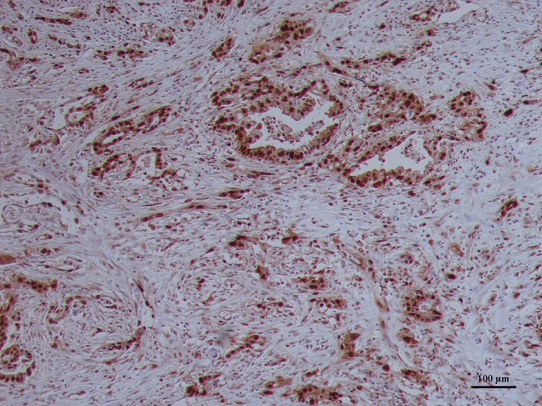


Patient 13

**No Recurrence (n=14)**

**
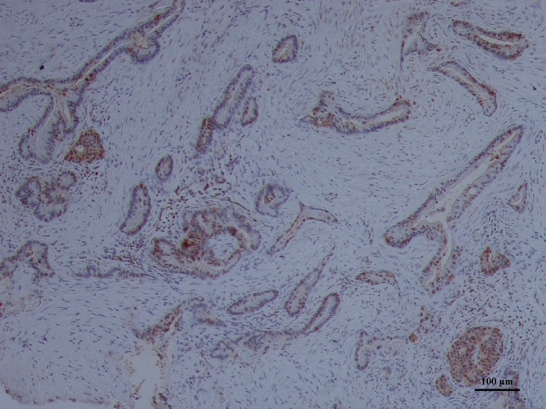

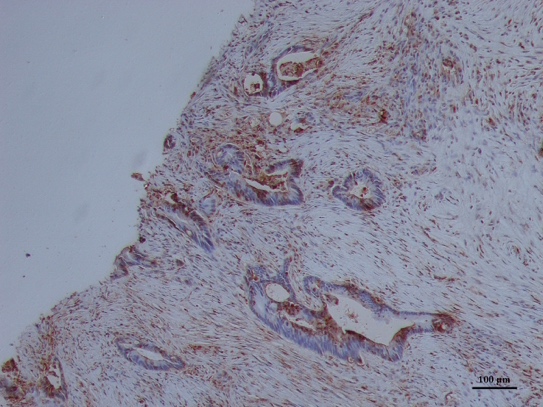
**

Patient 1 Patient 2

**
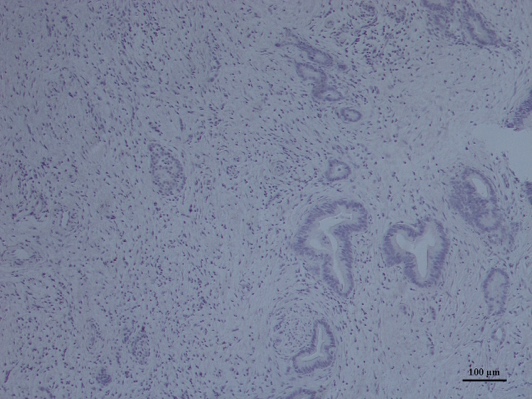

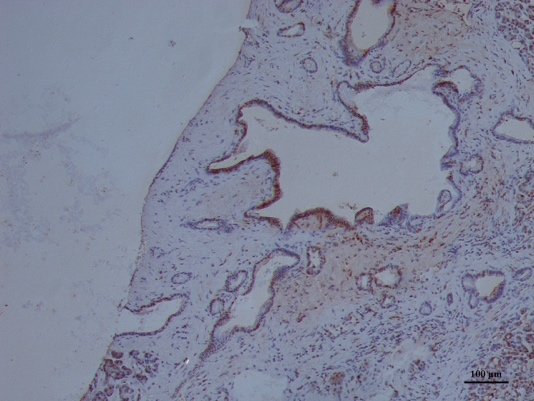
**

Patient 3 Patient 4

**
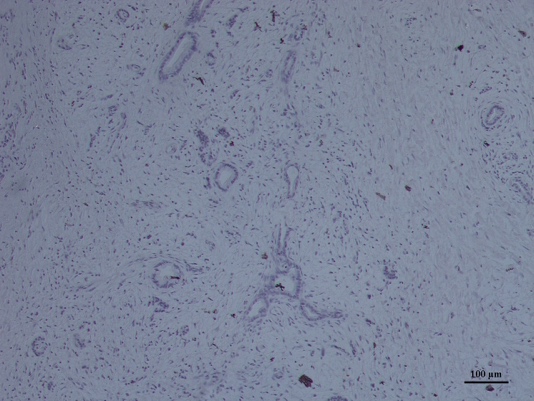

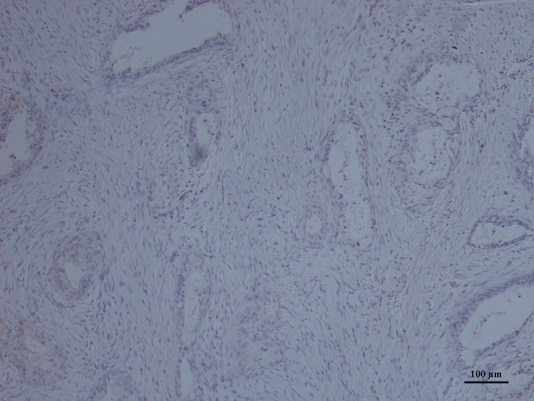
**

Patient 5 Patient 6

**
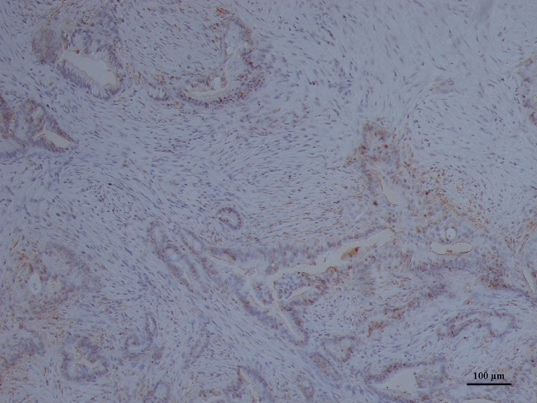

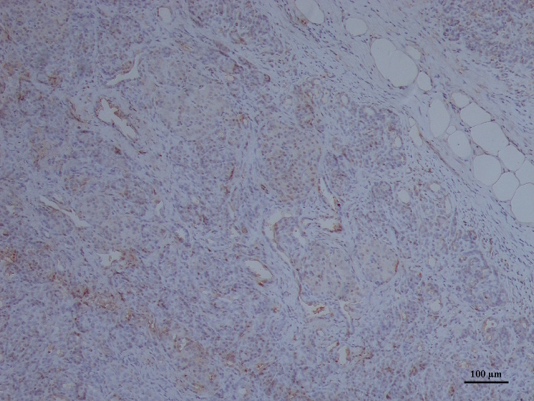
**

Patient 7 Patient 8

**
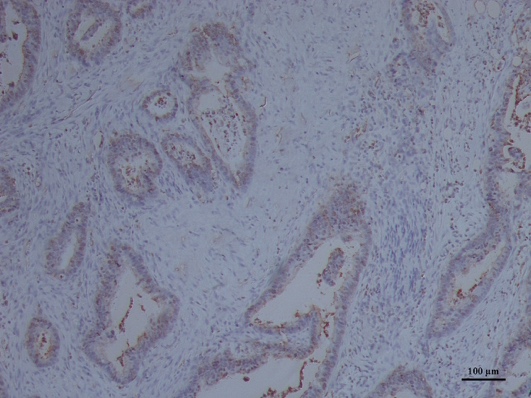

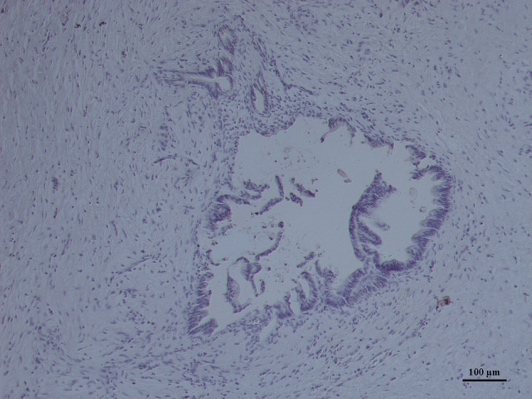
**

Patient 9 Patient 10

**
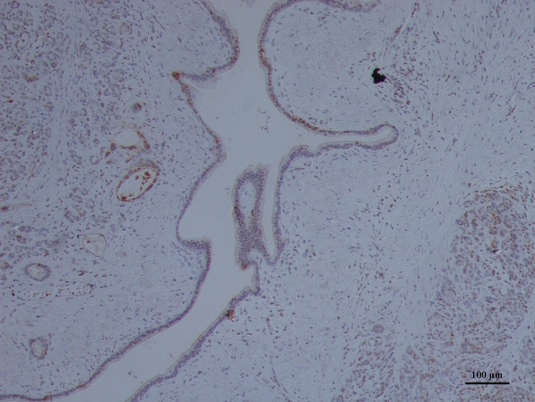

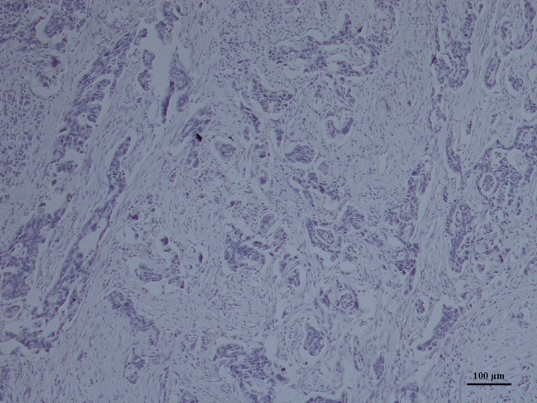
**

Patient 11 Patient 12

**
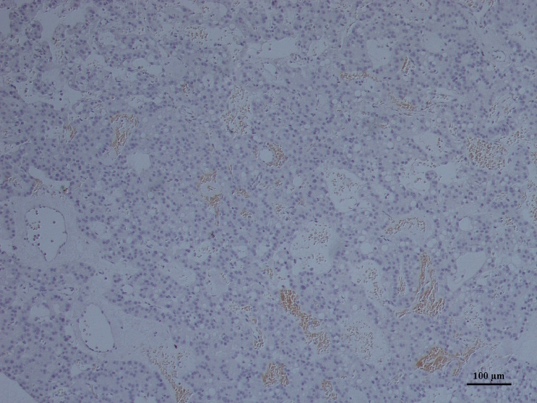

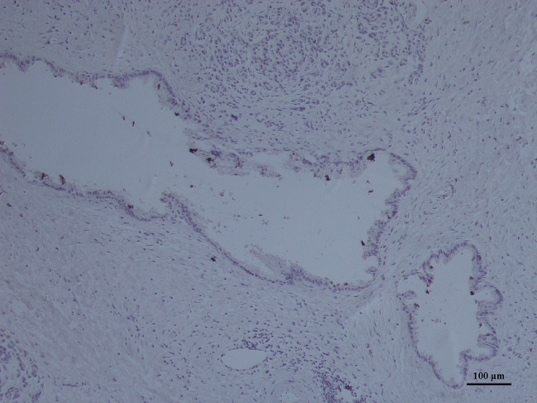
**

Patient 13 Patient 14

**CD4**

**Postoperative Liver Metastasis (n=13)**


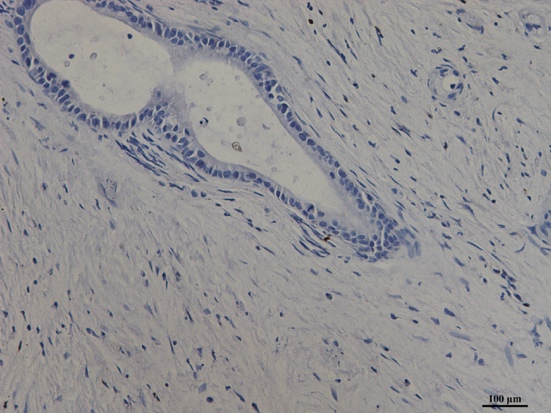

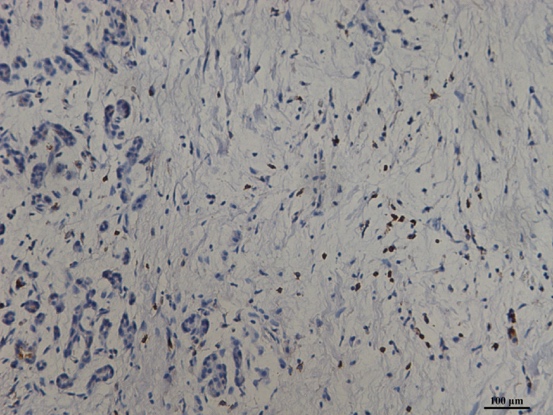


Patient 1 Patient 2


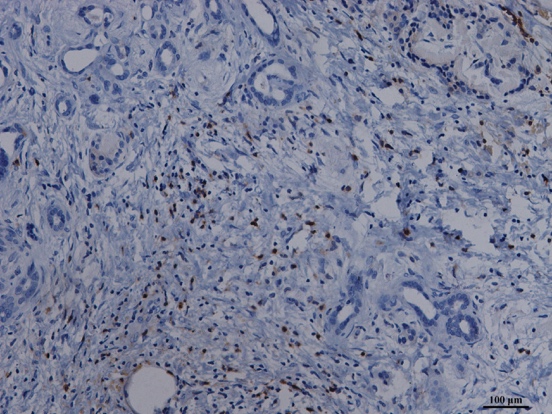

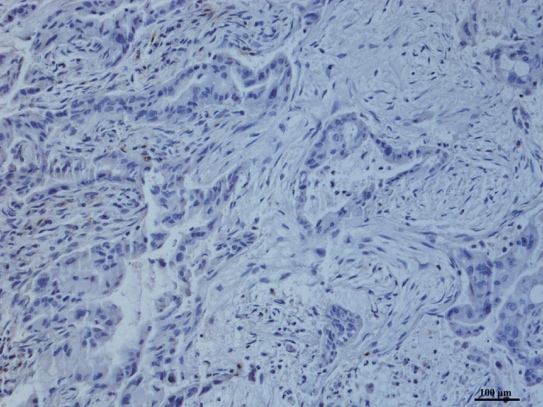


Patient 3 Patient 4


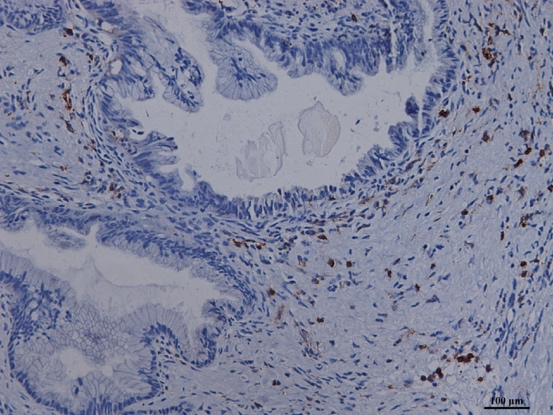

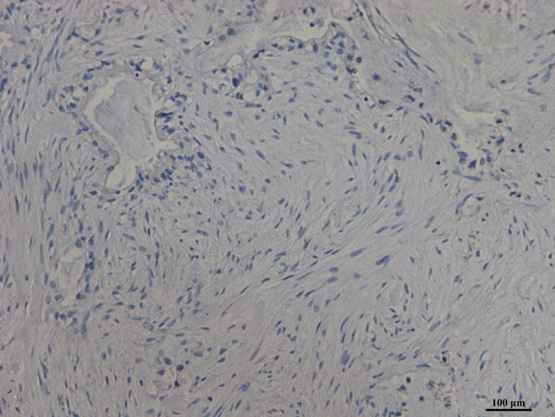


Patient 5 Patient 6


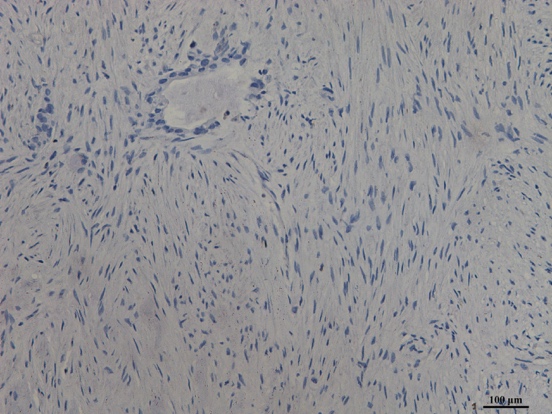

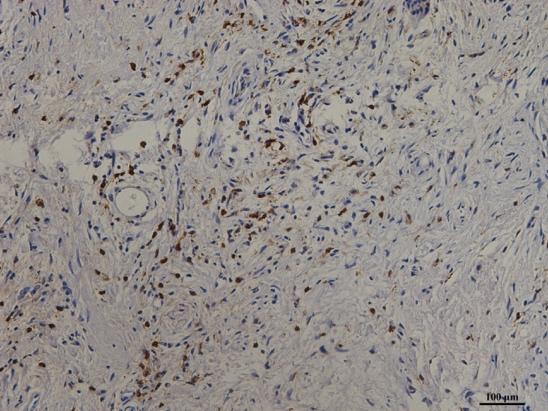


Patient 7 Patient 8


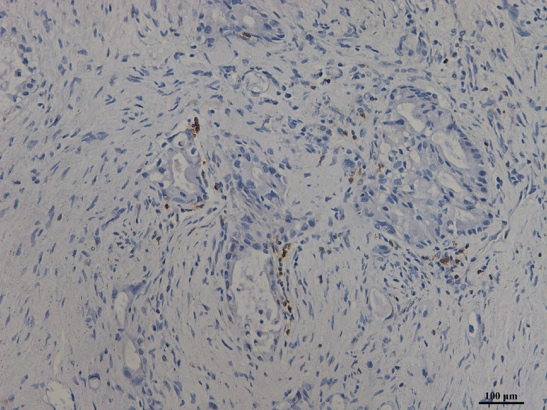

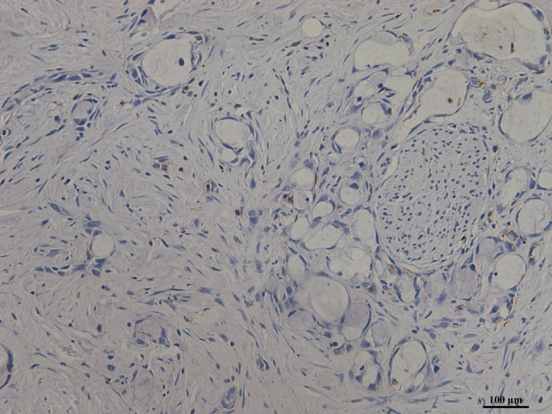


Patient 9 Patient 10


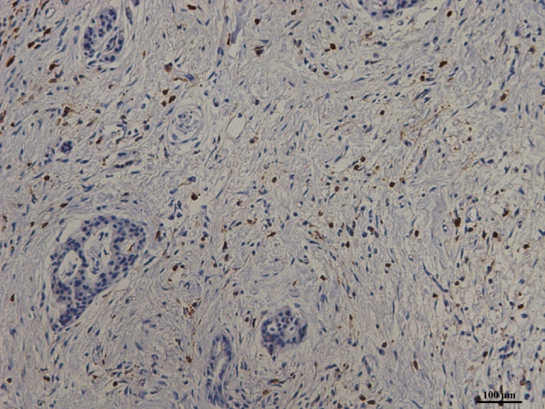

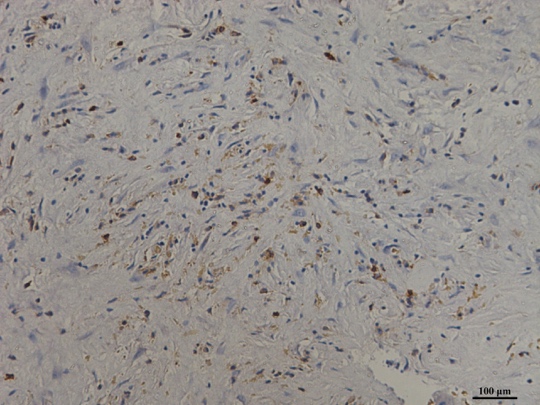


Patient 11 Patient 12


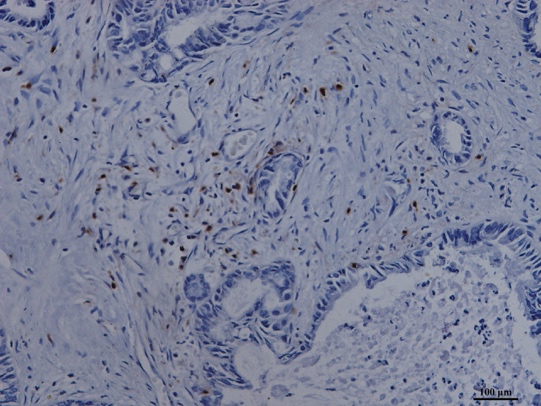


Patient 13

**No Recurrence (n=14)**

**
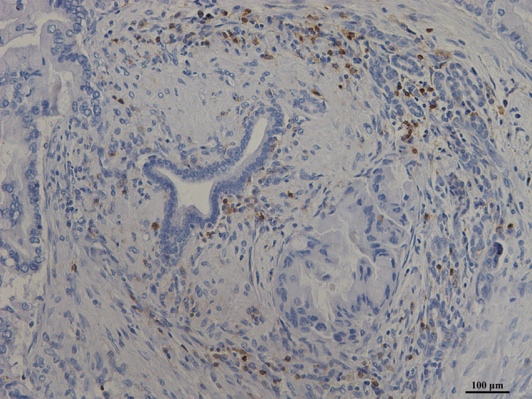

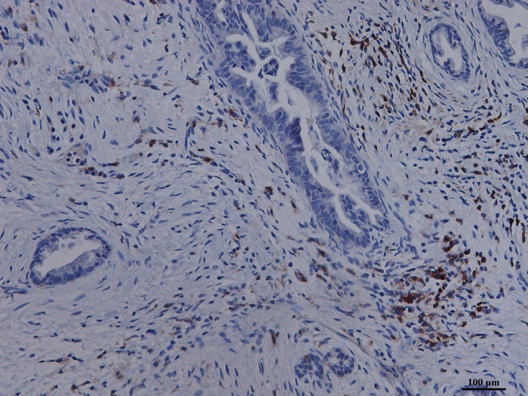
**

Patient 1 Patient 2

**
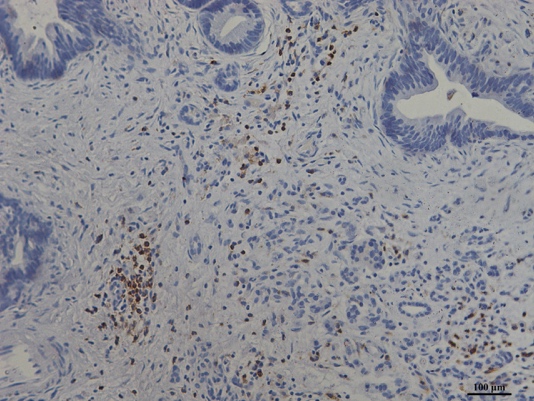

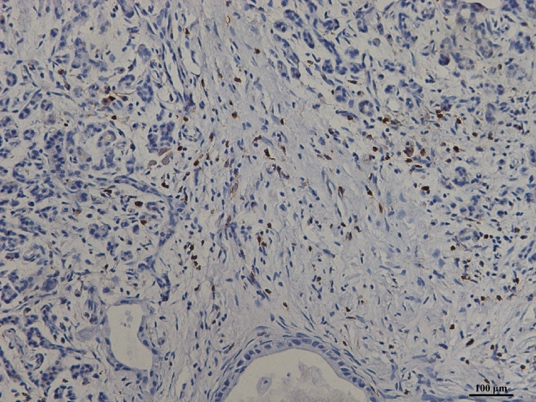
**

Patient 3 Patient 4

**
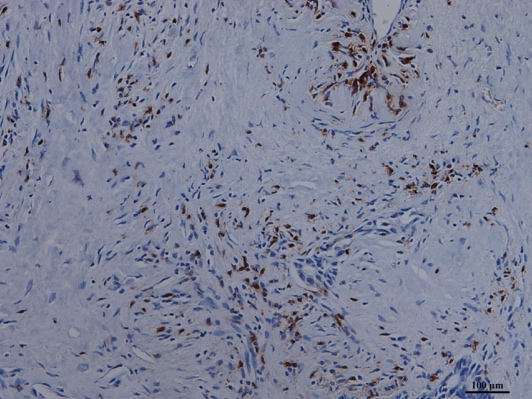

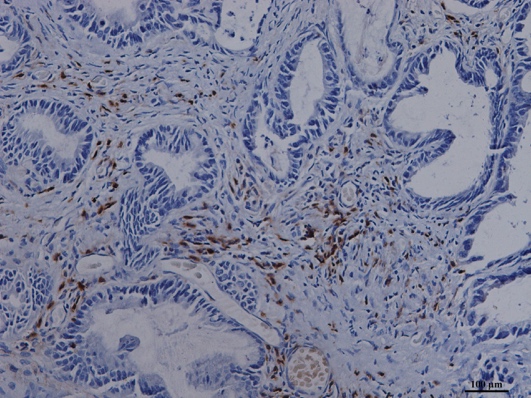
**

Patient 5 Patient 6

**
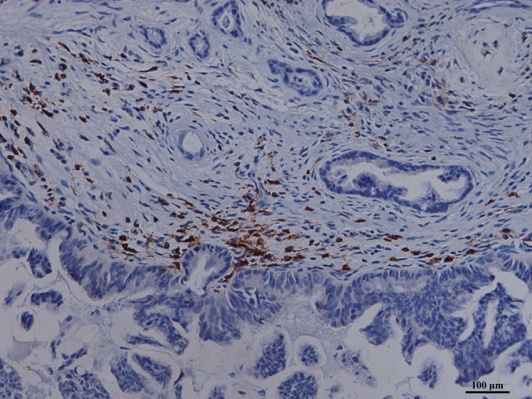

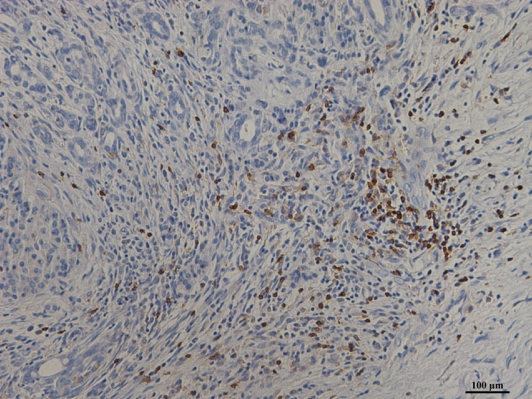
**

Patient 7 Patient 8

**
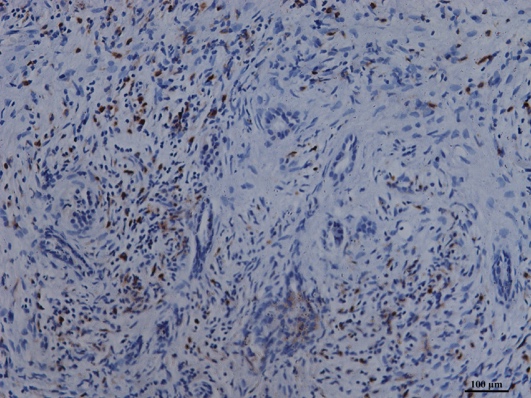

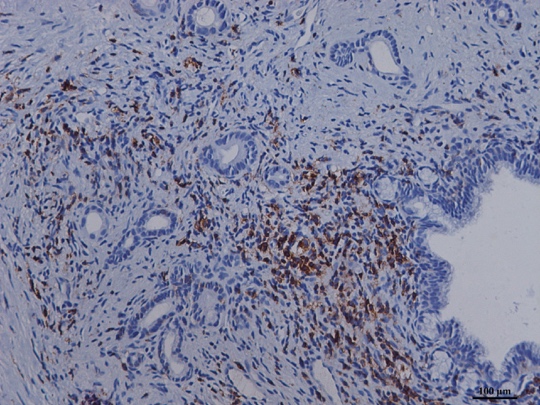
**

Patient 9 Patient 10

**
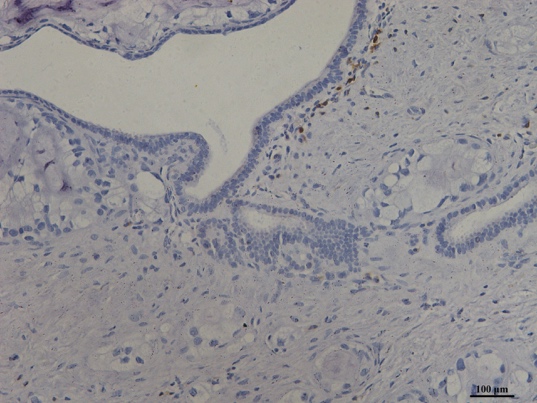

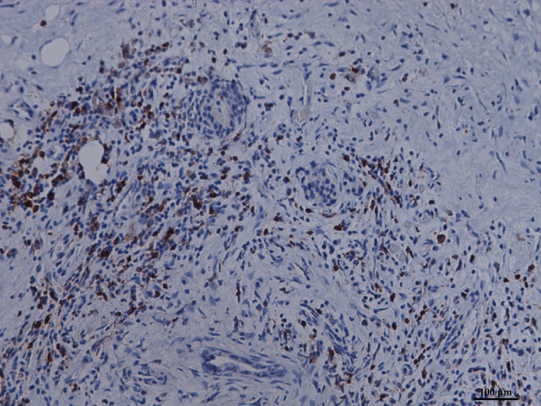
**

Patient 11 Patient 12

**
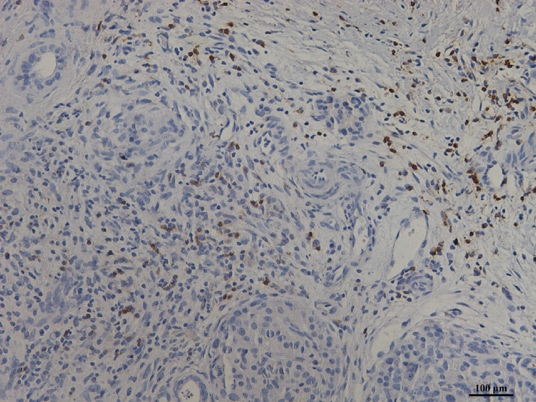

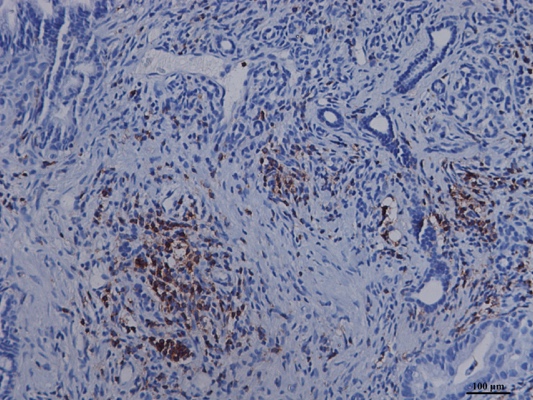
**

Patient 13 Patient 14

**TCGA list (Postoperative Liver Metastasis)**

TCGA-2J-AAB6. TCGA-2J-AAB9. TCGA-2J-AABO. TCGA-2J-AABR. TCGA-2J-AABU

TCGA-2J-AABV. TCGA-3A-A9I9. TCGA-3A-A9IU. TCGA-F2-7273. TCGA-FB-A545

TCGA-FB-A5VM. TCGA-FB-AAQ2. TCGA-FZ-5919. TCGA-H6-8124. TCGA-HZ-7918

TCGA-HZ-7924. TCGA-HZ-8003. TCGA-IB-7645. TCGA-IB-7646. TCGA-IB-7647

TCGA-IB-7649. TCGA-IB-7890. TCGA-IB-7897. TCGA-IB-A5SS. TCGA-IB-A6UF

TCGA-IB-A7LX. TCGA-IB-AAUO. TCGA-L1-A7W4. TCGA-OE-A75W. TCGA-PZ-A5RE

TCGA-RB-AA9M. TCGA-US-A774. TCGA-XD-AAUI

**TCGA list (No Recurrence)**

TCGA-2J-AAB8. TCGA-Q3-AA2A. TCGA-HZ-A4BH. TCGA-IB-AAUS. TCGA-FB-AAPS

TCGA-HV-A5A4. TCGA-IB-AAUU. TCGA-HV-A5A5. TCGA-F2-6880. TCGA-HZ-7923

TCGA-2J-AABT. TCGA-YB-A89D. TCGA-LB-A8F3. TCGA-IB-AAUV. TCGA-Q3-A5QY

TCGA-XD-AAUG. TCGA-IB-AAUP. TCGA-Z5-AAPL. TCGA-IB-A7M4. TCGA-2J-AABK

TCGA-HZ-A49H. TCGA-XD-AAUL. TCGA-F2-A8YN. TCGA-F2-A44H. TCGA-IB-A5ST

TCGA-HZ-A49G. TCGA-2J-AABE. TCGA-HZ-8001. TCGA-S4-A8RM. TCGA-3A-A9IX

TCGA-IB-7652. TCGA-IB-7885. TCGA-3A-A9I5. TCGA-YY-A8LH
